# High variability and non-neutral evolution of the mammalian *avpr1a *gene

**DOI:** 10.1186/1471-2148-7-176

**Published:** 2007-09-27

**Authors:** Sabine Fink, Laurent Excoffier, Gerald Heckel

**Affiliations:** 1 Computational and Molecular Population Genetics (CMPG), Zoological Institute, University of Bern, Baltzerstrasse 6, CH-3012 Bern, Switzerland

## Abstract

**Background:**

The arginine-vasopressin 1a receptor has been identified as a key determinant for social behaviour in *Microtus *voles, humans and other mammals. Nevertheless, the genetic bases of complex phenotypic traits like differences in social and mating behaviour among species and individuals remain largely unknown. Contrary to previous studies focusing on differences in the promotor region of the gene, we investigate here the level of functional variation in the coding region (exon 1) of this locus.

**Results:**

We detected high sequence diversity between higher mammalian taxa as well as between species of the genus *Microtus*. This includes length variation and radical amino acid changes, as well as the presence of distinct protein variants within individuals. Additionally, negative selection prevails on most parts of the first exon of the *arginine-vasopressin receptor 1a (avpr1a) *gene but it contains regions with higher rates of change that harbour positively selected sites. Synonymous and non-synonymous substitution rates in the *avpr1a *gene are not exceptional compared to other genes, but they exceed those found in related hormone receptors with similar functions.

**Discussion:**

These results stress the importance of considering variation in the coding sequence of *avpr1a *in regards to associations with life history traits (e.g. social behaviour, mating system, habitat requirements) of voles, other mammals and humans in particular.

## Background

The genetic bases of complex phenotypic traits like differences in social and mating behaviour among species and individuals remain largely unknown [1]. Most such traits are probably under polygenic control and the contribution of each gene to the phenotype is often very difficult to assess [2]. Even for genes with large effects, it is highly challenging to identify the causes of particular phenotypic differences because genetic variation is rarely restricted to dichotomous polymorphism in a gene [e.g. [3-6]]. Genetic variation at a locus is not only shaped by locus- or site-specific selective processes but also by the evolutionary history of the particular species or population.

One of the best examples of a single gene with large effects involved in very specific phenotypic and behavioural differences is the *arginine-vasopressin receptor 1a *(*avpr1a*). This gene has been proposed to play a key role in controlling variation in mammalian social behaviour [7-11], and it has been particularly well-studied for its role in the formation of mating systems in rodents from the genus *Microtus *[12-15]. Phenotypic differences between species in arginine-vasopressin 1a receptor (V1aR) distribution in the brain and contrasting social behaviour were largely attributed to the presence of a repetitive expansion in the regulatory region upstream of the gene [12-15]. The transfer of the entire *avpr1a *gene region including the repetitive expansion or the coding region from a monogamous vole to non-monogamous voles and other rodents resulted in modified V1aR distributions and changes in social behaviour [12]. Additionally, monogamous voles showed increased affiliative behaviour (measured as time spent in contact with other voles, see in [14]) after injection of the arginine-vasopressin (AVP) hormone in the brain, while non-monogamous voles displayed unchanged social behaviour [14]. However, AVP has two main roles: it controls higher cognitive functions such as memory and learning in the brain, and it acts peripherally by facilitating water absorption in the kidney and by contracting smooth muscle cells from blood vessels [16]. The impact of hormones on behavioural variation may therefore also depend on environmental conditions [see [17]]. A recent study showed further that neither social nor genetic monogamy are strictly associated with the presence of the repetitive expansion in the regulatory region of *avpr1a *in voles and other mammals [18].

In contrast to polymorphism in the regulatory region of *avpr1a*, variation in the coding part is assumed to be low and functionally negligible [14,19,20]. Rodent *avpr1a *patterns have been proposed as mammalian model systems for the study of the role of hormone receptors in the formation of complex social interactions, including human social disorders [21]. Studies of human *avpr1a *have mainly focused on variation in the non-coding upstream region of the gene, and associations have been reported with autism [22-24], eating behaviour [25], self perception [11] and even creative dance performance [26]. Single nucleotide polymorphisms in the human *avpr1a *gene have been detected [22-24,27], but it is unknown if they affect the encoded protein. Previous studies of the Microtine *avpr1a *have not explicitly studied levels and patterns of variation in the coding region at the inter- or intra-specific level. Its potential influence on social behaviour and interactions in voles and other mammals remains therefore totally unknown.

We use here an evolutionary approach to investigate variation in parts of the coding region of the mammalian *avpr1a *gene. We analyse patterns of nucleotide and amino acid (AA) polymorphism in the *Microtus *genus represented by 24 species from three continents (Europe, North America, Asia), and compare it to the *avpr1a *diversity found in various higher mammalian taxa. Furthermore, we examine rate variation among the functionally important regions – the ligand binding site or the G-protein binding domain [16] – and other parts of the V1aR, and we test for the role of selection in shaping variability in the *avpr1a *gene.

## Results

### Microtine *avpr1a *diversity

The analysis of a large fragment (792 bp) of the first exon (total 970 bp) of the *avpr1a *gene in the genus *Microtus *revealed unexpectedly high levels of variation with an overall nucleotide diversity of 0.0161. The sequencing of individuals from 24 species revealed 12 heterozygous individuals, while the two individuals obtained from GenBank were apparently homozygous for *avpr1a*. After cloning of heterozygous PCR-products, a total of 36 different sequences were detected among the 48 chromosomes, which showed overall 103 variable positions (13%). Despite this large amount of diversity, one chromosome sequence (E01, see Table 1) was identical between two individuals of different species (*M. tatricus, M. oeconomus*).

**Table 1:** Origin of rodent samples and *avpr1a *sequences

***Microtus *species**	**continent**	**country**	**locality**	**chromosome label**	**accession number**
*tatricus*	Europe	Slovakia	High Tatra Mountains	E01	EU176005
*agrestis*	Europe	Finland	Lapua	E02	EU175968
				E03	EU175969
*arvalis*	Europe	Switzerland	Belp	E04	EU175970
				E05	EU175971
*rossiaemeridionalis*	Europe	Macedonia	Gradsko	E06	EU175972
				E07	EU175973
*multiplex*	Europe	Switzerland	Ticino	E08	EU175974
*nivalis*	Europe	Spain	Avila	E09	EU175975
*felteni*	Europe	Greece	Thessalia	E10	EU175976
*thomasi*	Europe	Greece	Nomos Arkadia	E11	EU175977
*cabrerae*	Europe	Portugal	Cauda	E12	EU175978
*schelkovnikovi*	Europe	Azerbaijan	Talysh	E13	EU175979
				E14	EU175980
*socialis*	Europe	Azerbaijan	Stepanakert	E15	EU175981
				E16	EU175982
*oeconomus*	North-America	Canada	Yukon	E01	EU176006
*ochrogaster*	Gene bank sequence			NA01	AF069304
*ochrogaster*	North-America	USA	Kansas	NA01	EU175983
*montanus*	Gene bank sequence			NA02	AF070010
*montanus*	North-America	USA	Missoula	NA03	EU175984
*pinetorum*	North-America	USA	Calloway	NA04	EU175985
				NA05	EU175986
*californicus*	North-America	USA	Stanislaus	NA06	EU175987
				NA07	EU175988
*chrotorrhinus*	North-America	USA	Minnesota	NA08	EU175989
				NA09	EU175990
*richardsoni*	North-America	USA	Minnesota	NA10	EU175991
*longicaudus*	North-America	USA	Sierra County	NA11	EU175992
				NA12	EU175993
*abbreviatus*	North-America	Alaska	Hall Island	NA13	EU175994
*oregoni*	North-America	USA	Oregon	NA14	EU175995
				NA15	EU175996
*townsendii*	North-America	USA	Oregon	NA16	EU175997
				NA17	EU175998
*montebelli*	Asia	Japan	Tottori lity	A01	EU175999
				A02	EU176000
*kikuchii*	Asia	Taiwan	Tao-Yuan	A03	EU176001

**other rodents:**					

*Arvicola terrestris*	Europe	Switzerland	Bern	A. terrestris	EU176002
*Apodemus sylvaticus*	Europe	Switzerland	Bern	A. sylvaticus	EU176003
*Clethrionomys glareolus*	Europe	Germany	Waldbeck	C. glareolus	EU176004

Sequences of heterozygous individuals were resolved by cloning and sequencing of PCR-products (see text). Chromosomes are labelled according to continent of origin of the samples (E = Europe, NA = North America, A = Asia). GenBank accession numbers are given for *avpr1a *nucleotide sequences.

**Figure 1:**
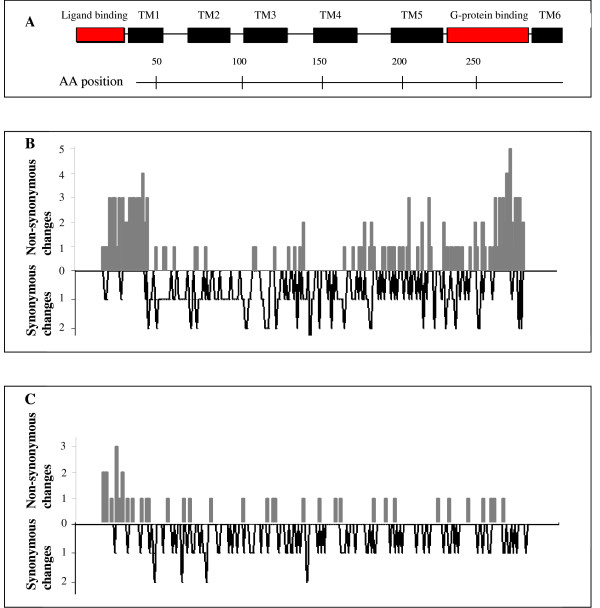
**Synonymous and non-synonymous changes in the *avpr1a *gene**. A: Schematic overview of the structure of V1a receptor adapted from a model of *Mus musculus *[95]. The functionally important receptor regions (ligand binding and G-protein binding domains) are shown in red, while six out of seven transmembrane regions are displayed in black (label TM1-TM6). B: Non-synonymous (grey bars) and synonymous (black lines) substitutions in Eutherian mammals and the marsupial *Monodelphis domestica *(one DNA sequence per species, see text). Highest numbers of non-synonymous substitutions are present in the ligand binding and the G-protein binding domains, while synonymous substitutions are scattered along the whole gene. C: Non-synonymous (grey bars) and synonymous (black lines) changes for 24 species of the *Microtus *genus (one sequence per species, see text). High numbers of AA variants are found in the ligand binding domain only, while the G-protein binding domain is relatively conserved. Similar to the pattern in higher mammalian taxa, synonymous substitutions are equally frequent along the exon.

**Figure 2:**
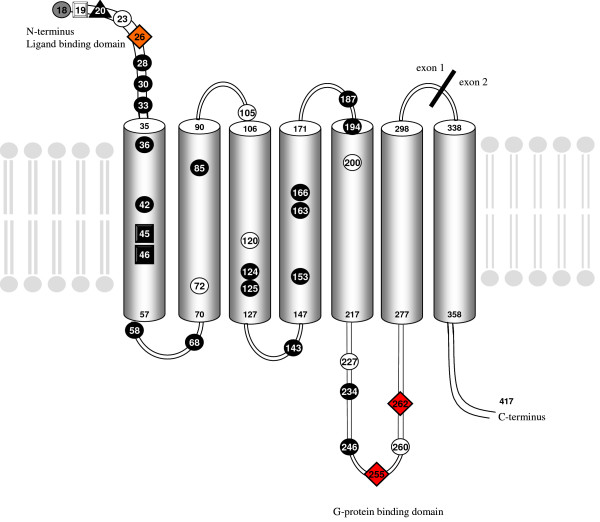
Structural model of the V1a receptor with amino acid substitutions in the genus *Microtus*. AA substitutions are spread over the whole protein, but largest numbers of changes are found in the functionally important ligand binding domain. Position of changes and type of changes are marked as: black circle = radical change; white circle = conservative change; grey circle = conservative and radical changes at the same position; white square = deletion; black square = insertion; black triangle = radical change and deletion at the same position. Changes between protein types within an individual occur in the functionally important regions (ligand and G-protein binding domains) and are marked as red diamond for a radical change, and as orange diamond for a conservative change.

After translation into AA, the 36 DNA sequence types coded for 24 different proteins. Individuals from three species remained heterozygous at the AA level (in *M. arvalis, M. rossiaemeridionalis *and *M. oregoni*). Two protein types were shared among several species (*M. longicaudus, M. socialis *and *M. tatricus, M. oeconomus, M. multiplex*, respectively). The highest number of AA variants was found in the ligand binding domain of the V1aR with up to four different AAs per position (Figure 1). The number of AA changes was significantly different between ligand binding domain, G-protein binding domain and transmembrane regions (χ^2 ^= 13.95, df = 2, p < 0.001). AA changes occurred at eight positions (24%) in the ligand-binding N-terminus of the protein, at six positions (18%) in the G-protein binding domain, and at 20 positions (58%) between these functionally important regions (Figure 2). Significantly more AA changes were present in the N-terminus (χ^2 ^= 13.92, df = 1, p < 0.001) than in the first five transmembrane regions or the G-protein binding domain (χ^2 ^= 13.92, df = 1, p < 0.001). The G-protein binding domain did not show significantly more AA substitutions than the transmembrane regions (χ^2 ^= 0.06, df = 1, p > 0.5). The individuals with two V1aR types differed at the intra-individual level either in the ligand binding domain (*M. oregoni*, position 26) or in the G-protein binding domain (*M. rossiaemeridionalis*, position 255; *M. arvalis*, position 262).

**Figure 3:**
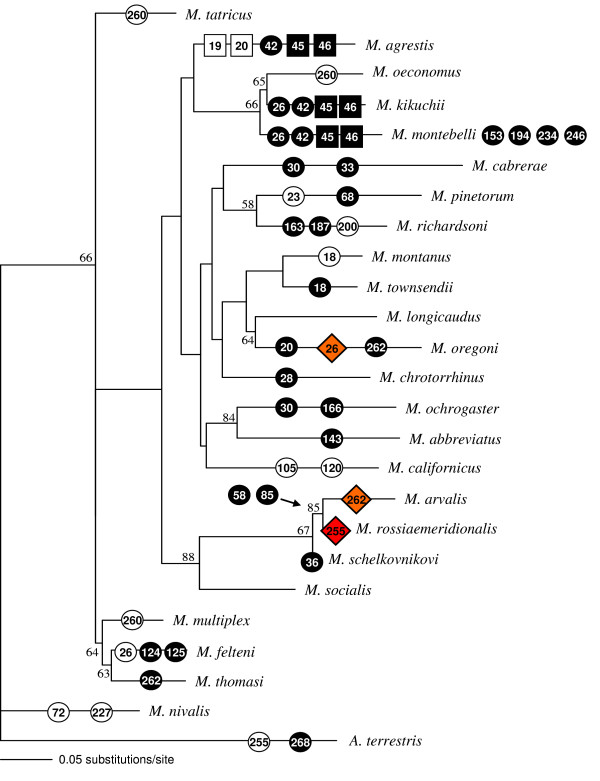
Amino acid alterations of the *avpr1a *gene plotted onto a mitochondrial cytochrome *b *phylogeny of the genus *Microtus*. Positions and types of changes are labelled as in Figure 2. Bootstrap values > 50 (10'000 replicates) of the maximum likelihood method are shown on the branches. AA alterations in *Microtus *segregate generally independently of the phylogenetic background except for the closely related sister species *M. arvalis *and *M. rossiaemeridionalis *which show two identical changes at the same positions (58, 85). Additionally, a two AA long insertion together with an alteration at position 42 appear in the cluster of *M. agrestis *together with *M. montebelli *and *M. kikuchii*, where the changes seem to have been subsequently lost in *M. oeconomus*.

Genetic variation within the *Microtus *genus included the deletion of two AAs in the ligand binding domain in one species (*M. agrestis*) and an insertion of two AA in the first transmembrane region in three species (*M. agrestis, M. montebelli, M. kikuchii*). AA insertions segregate together with an AA change at position 42 for a group of related species (Figure 3). These protein alterations were apparently subsequently lost in one species of this cluster *(M. oeconomus*, see Figure 3). The two closely related sister species *M. arvalis *and *M. rossiaemeridionalis *[28] share two AA changes (58, 85), while otherwise protein types did not obviously segregate with phylogenetic relationships as inferred from the mitochondrial cytochrome *b *gene.

23 AA substitutions involved radical (at least one change between physico-chemical classes considering polarity, charge and volume; see in [29]) and 10 conservative (all three categories reveal the same physico-chemical characteristics for the interchangeable AAs) changes. Ten of the radical changes were found in the ligand binding and the G-protein binding domains (see Figure 2).

Different selection tests detected mostly negative selection on the Microtine *avpr1a *gene and some ambiguous evidence for positive selection on particular parts. HyPhy detected no signal of positive selection and several sites under negative selection between the functionally important regions (codon positions 50, 67, 68, 82, 170, 186, p < 0.05). PAML results suggested equal substitution rates among *Microtus *lineages (M0 vs M3: 2Δ*l *= 6.1684, 4 df, p > 0.05, see Table 2), and no statistical support for positive selection in any part of the gene (M1 vs M2: 2Δ*l *= 0, 2 df, p > 0.5; M7 vs M8: 2Δ*l *= 0, 2 df, p > 0.5). Nevertheless, some codon positions had an ω exceeding 1 in the analysis of models for positive selection (M2 and M8: positions 18 and 26; M8 only: position 30), and these sites lie in the ligand binding domain at the N-terminus of the gene. These codons were positively selected against a background of strong purifying selection acting on 96% of the sites, which is in agreement with the results of HyPhy.

**Table 2:** Results of *avpr1a *selection tests performed with the software PAML

**model**	**parameters**	**likelihood *l***	**positively selected sites**
**A: *Microtus***			
M0, one ratio		-1970.4456	Not allowed
M1, neutral	*p*_0 _= 0.9600, ω_0 _= 0.0602	-1967.7956	Not allowed
	*p*_1 _= 0.0400, ω_1 _= 1		
M2, selection	*p*_0 _= 0.9600, ω_0 _= 0.0602	-1967.7956	18,26
	*p*_1 _= 0.0199, ω_1 _= 1		
	*p*_2 _= 0.0200, ω_2 _= 1		
M3, discrete	*p*_0 _= 0.1311, ω_0 _= 0.0406	-1967.3614	
	*p*_1 _= 0.7474, ω_1 _= 0.0407		
	*p*_2 _= 0.1215, ω_2 _= 0.4609		
M7, beta	p = 0.29056, q = 2.75313	-1967.4361	Not allowed
M8, beta and ω	*p*_0 _= 1.0000, p = 0.29056, q = 2.75313	-1967.4361	18,26,30
	*p*_1 _= 0.0000, ω = 1.0000		
**B: Mammals**			
M0, one ratio		-3628.0022	Not allowed
M1, neutral	*p*_0 _= 0.8433, ω_0 _= 0.0452	-3567.8186	Not allowed
	*p*_1 _= 0.1568, ω_1 _= 1		
M2, selection	*p*_0 _= 0.8433, ω_0 _= 0.0452	-3567.8186	
	*p*_1 _= 0.1356, ω_1 _= 1		
	*p*_2 _= 0.0212, ω_2 _= 1		
M3, discrete	*p*_0 _= 0.5084, ω_0 _= 0.0000	-3538.7336	
	*p*_1 _= 0.3169, ω_1 _= 0.1063		
	*p*_2 _= 0.1747, ω_2 _= 0.4661		
M7, beta	p = 0.23059, q = 1.71469	-3540.0533	Not allowed
M8, beta and ω	*p*_0 _= 1.0000, p = 0.23059, q = 1.71466	-3540.0535	25,36
	*p*_1 _= 0.0000, ω = 2.02300		
**Branch specific models:**			
MA, foreground branch = ***M. montanus***		-3567.8186	none
MA, foreground branch = ***A. terrestris***		-3567.8187	none
MA, foreground branch = ***C. glareolus***		-3567.8186	none
MA, foreground branch = ***A. sylvaticus***		-3567.8186	none
MA, foreground branch = ***M. musculus***		-3567.3630	none
MA, foreground branch = ***R. norvegicus***		-3567.8186	none
MA, foreground branch = ***O. aries***		-3563.2582	191,228,231,243,247,260
MA, foreground branch = ***B. taurus***		-3567.3824	243
MA, foreground branch = ***C. familiaris***		-3567.8186	191
MA, foreground branch = ***M. mulatta***		-3567.8186	none
MA, foreground branch = ***P. troglodytes***		-3567.5837	247
MA, foreground branch = ***H. sapiens***		-3567.5242	259,274
MA, foreground branch = ***M. domestica***		-3567.7818	none

Maximum likelihood methods were applied to compare models allowing for positive or negative selection (M2, M3, M8 and MA) with models without selection (M0, M1, M7). Parameters are represented as *p*_0 _= proportion of sites where ω < 1 (ω_0_), *p*_1 _= proportion of sites where ω = 1 (ω_1_), and for models with selection *p*_2 _= proportion of sites where ω > 1 (ω_2_). For models M7 and M8, p and q represent parameters of the beta distribution. The log likelihood *l *of each model is given as well as the position of positively selected codons (where ω > 1). **A**: Overall selection tests in *Microtus *species (M1 vs. M2, M7 vs. M8) and ratio heterogeneity (M0 vs. M3).**B**: Overall selection tests of *avpr1a *and tests for positive selection along specific mammal branches (M1 vs. MA).

### Mammalian *avpr1a *diversity

**Figure 4:**
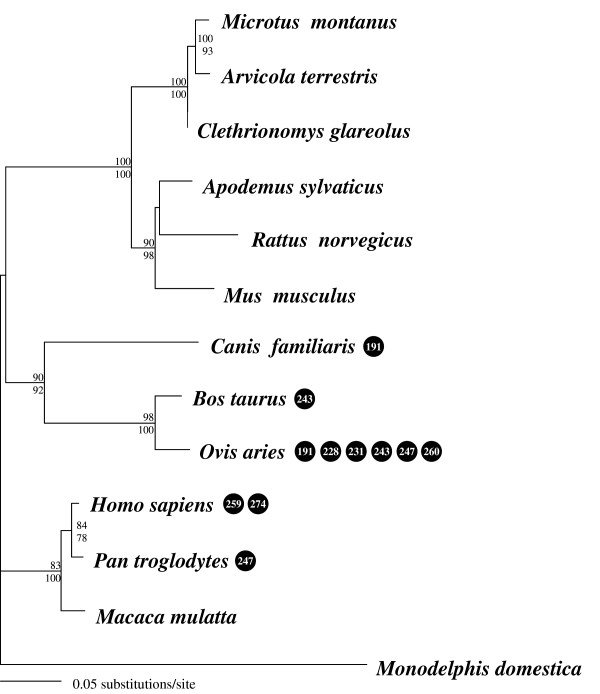
Maximum likelihood tree inferred from the nucleotide sequences of exon 1 of the *arginine-vasopressin 1a receptor *gene for various mammalian taxa. Bootstrap values > 50 are shown for the maximum likelihood method above branches and for neighbour-joining below branches. Positively selected sites (ω > 1) are shown in black circles. Note that most of these sites are found in the G-protein binding domain (231–274). Only two positively selected sites (191; 228) were detected outside this domain in two species (*O. aries *and *C. familiaris*).

To contrast Microtine *avpr1a *diversity to variability in other mammals, we sequenced the corresponding fragment of the first exon in different rodent taxa (*Arvicola terrestris*, *Clethrionomys glareolus, Apodemus sylvaticus*, see Table 1) and supplemented it with published nucleotide sequences of several Eutherian mammals, as well as a marsupial sequence (*Monodelphis domestica*) as outgroup. Nucleotide sequence analyses revealed high nucleotide diversity (0.1488) and a high proportion of variable positions (41.7%; 36.38% without marsupial). A phylogenetic tree based on nucleotide sequences using ML and NJ reconstruction methods revealed the same topology (Figure 4), with e.g. rodents and primates forming highly supported clades.

The high diversity found at the nucleotide level resulted in high AA diversity after translation with all species showing unique AA sequence types. Most changes occurred in the two functionally important regions of the V1aR: the ligand binding domain and the G-protein binding domain (Figure 1). The latter region included many AA deletions and insertions, resulting in length variation among mammals. Except for a 3 AA long deletion in several rodents (*M. montanus, A. terrestris, C. glareolus*), the other insertions and deletions occurred in single species only.

**Figure 5:**
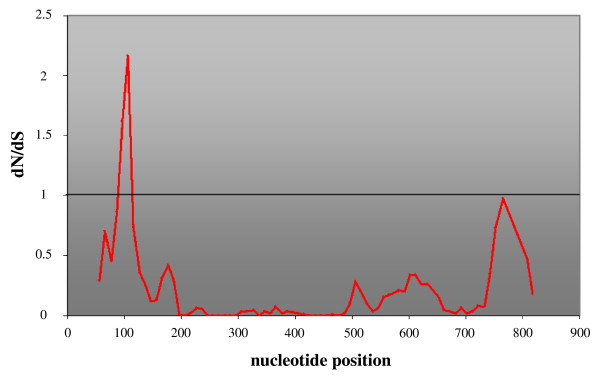
Sliding window analysis of the ratio of non-synonymous substitutions (d_N_) over synonymous substitutions (d_S_) along the *avpr1a *gene of mammals compared to the marsupial *Monodelphis domestica *(see text). The ratio is drawn over the midpoint window position (window size 30, step size 10) from nucleotide position 50 to 800 from the start codon (due to primer selection). d_N_/d_S _exceeds 1 in the ligand binding domain, which indicates positive selection in this region. A second peak of d_N_/d_S _close to 1 is found around 750 bp corresponding to the G-protein binding domain of the AVP 1a receptor.

Sliding window analyses of d_N_/d_S _ratios along the gene showed a strong signal of positive selection in the ligand binding domain (d_N_/d_S _= 2.163), while the d_N _and d_S _values in the region around the G-protein binding domain are equal due to relatively more synonymous variation (Figures 1; 5). The transmembrane regions show comparatively few non-synonymous mutations (Figure 1) which leads to small d_N_/d_S _ratios (Figure 5).

Despite the evidence for positive selection on the ligand binding domain, further tests rather suggested generally negative selection on *avpr1a*. PAML detected significant rate variation among the lineages (M0 vs M3: 2Δ*l *= 178.537, df = 4 p < 0.05), where 88% of all sites were under strong purifying selection, while 12% showed relaxed purifying selection acting on these sites (Table 2). PAML revealed no evidence for positive selection overall (M1 vs M2: 2Δ*l *= 0, df = 2 p > 0.05; M7 vs M8:2Δ*l *= 0.0004, df = 2 p > 0.05; Table 2). HyPhy detected five negatively selected sites in functionally important regions and 24 in-between (codon positions 33, 41, 47, 48, 52, 69, 71, 77, 87, 107, 119, 120, 125, 136, 138, 146, 152, 159, 178, 184, 198, 216, 223, 227, 230, 250, 251, 254, 279).

Considering the phylogenetic background of the species provided further evidence for non-neutral evolution of the *avpr1a *gene. For the mammalian branches, evolutionary models allowing for selection (MA) were not significantly better than models not incorporating selection (M1; see likelihood values Table 2). Codons with d_N_/d_S _ratios exceeding 1 were detected mainly in the G-protein binding domain (231–274), with only two species showing positively selected sites outside (*O. aries, C. familiaris*; positions 191, 228; see Figure 4).

**Figure 6:**
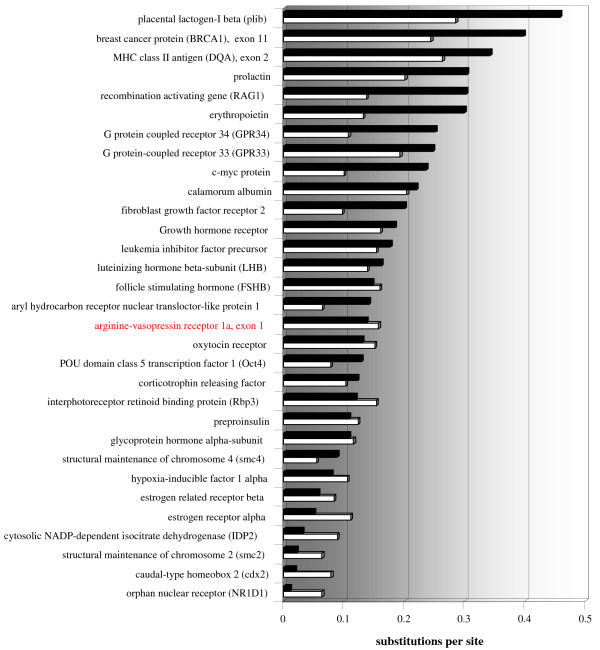
Comparison of synonymous and non-synonymous substitutions per site for orthologous nuclear genes in *Microtus*, mouse and rat. Genes are ranked according to non-synonymous substitutions (black bars) per site. Synonymous substitutions per site are shown as white bars.

Despite high variability among mammals in general and within *Microtus*, substitution rates of *avpr1a *are not exceptionally high relative to other nuclear genes in the comparison of mouse, rat and *Microtus *(Figure 6). Many genes investigated to date show much higher non-synonymous rates than *avpr1a *and synonymous rates rank this receptor gene only slightly higher. However, it is worth noting that substitution rates of *avpr1a *are higher than for other hormone receptors with related function like oxytocin, corticothropin or estrogen.

## Discussion

Our analyses of the *avpr1a *gene, shown to have high behavioural impact in the genus *Microtus *as well as in other mammals [12,30], revealed high nucleotide and protein diversity. Variation within *Microtus *involved many radical physico-chemical amino acid substitutions and deletions, which were located at functionally important regions of the V1aR. The pattern indicates positive selection on few codons in the ligand binding domain and possibly in the G-protein binding domain, but purifying selection on the majority of the gene.

### High genetic variation in the *avpr1a *gene

Genetic variability in the coding region of the *avpr1a *gene appears much higher and evolutionarily much more important than previously suggested [30,31]. DNA sequences of just two *M. ochrogaster *and *M. montanus *individuals were taken as evidence that the Microtine *avpr1a *gene was highly conserved [14,20,31]. However, our analyses reveal not only high levels of genetic variation in the coding region between mammalian species, but also within the genus *Microtus*. We detected up to 23 polymorphic positions in the first exon of the gene within a single *Microtus *individual compared to other closely related species, whereas studies of human *avpr1a *revealed a few synonymous and non-synonymous SNP in humans [22,27]. Population data from at least one *Microtus *species will be necessary to allow more detailed comparisons with human variation. However, the apparent difference between voles and humans may be explained in part by the longer evolutionary history of *Microtus *voles of at least several hundred thousand years with about three generations per year [28] and generally elevated mutation rates in rodents compared to primates [32,33]

Many AA replacements in the Microtine *avpr1a *gene involved radical physico-chemical changes, and several vole species showed deletions and insertions of AAs in this hormone receptor gene. Such length-variation in coding DNA within or among closely related species is remarkable, because it is usually restricted to non-coding DNA, where it may influence in particular cases the expression of genes but is generally functionally and selectively neutral [34-37]. Additionally, we detected considerable length variation in the G-protein binding region between different mammalian species. The diversity found among mammals might influence signal transduction, since already single AA changes can lead to differences in receptor activation in this region [16].

Amino acid positions identified as crucial for either ligand binding or G-protein activation in humans, mouse and rat are mainly conserved among *Microtus *species as well as among other mammals. A highly conserved triplet (Asp^148^-Arg^149^-Tyr^150^) with a role in signal transduction in many G-protein coupled receptors was conserved across all individuals analysed [38]. Additionally, a glycosylation site (Asn^27^) with a crucial role in protein folding or stabilization [39] remained conserved among all mammals and voles. Glu^185^, supposed to be involved in agonist and peptide as well as non-peptide antagonist binding to the V1aR [16], was highly conserved among mammals except for one *Microtus *individual (*M. richardsoni*), which showed a mutation to His^185^. This alteration together with an additional mutation at a glycosylation site (Asn^198^->Thr^198^) could lead to dysfunctions [39]. It is unclear if this would apply to voles since analyses of the specific roles of these sites in *Microtus *are lacking.

The observed high level of diversity and the detection of indels are unlikely to be due to gene duplications of *avpr1a *or the occurrence of pseudo-genes. *avpr1a *is a single copy gene in humans [40] and in rat [41], and a duplication has been found only in *M. ochrogaster *[14]. We cannot exclude that some of the detected variation stems from the presence of very recently duplicated sequences in some species. However, contrary to the truncated and clearly divergent version of *avpr1a *in *M. ochrogaster*, we found no indication for non-functionality in any of the sequences, such as reading frame shifts due to insertions or deletions of single nucleotides or the presence of premature stop codons [42]. This suggests that at least the majority of *avpr1a *gene variants is functional.

The potential functional relevance of variation in Microtine *avpr1a *is further emphasized by the detection of alleles coding for different protein types within the same individuals. It is worth noting that this involved again the ligand and G-protein binding domains. However, it is currently unclear if heterozygous individuals express different protein variants in the same tissue or if there is tissue specific expression [see [15]]. Gene expression studies are needed to investigate this further, since some receptor functions can as well be substituted by other receptors for hormones which are involved in very similar pathways [30,43]. This distinction would be of pharmaceutical importance for human health [see [44,45]]. Given the high variability in protein types, *Microtus *voles could serve as ideal models to study the processing and the expression of *avpr1a *gene products and their functional consequences in homo- and heterozygous individuals since they can be bred in the laboratory [21].

We further hypothesize that variation in the coding sequence of *avpr1a *might be related to life history traits (e.g. mating system, habitat), given the peripheral role of the V1aR in water retention in the kidney [16], and the social relevance when expressed in the brain [7-10]. Although kidney inefficiency was suggested as a major reason for the restriction of some *Microtus *species to moist habitat [46], we could not detect any connection between receptor type and a given habitat. Species occupying dry habitats (*e.g. M. multiplex, M. arvalis, M. lusitanicus, M. pinetorum, M. nivalis*, [47-50]) or wet habitats (e.g. *M. agrestis, M. rossiaemeridionalis, M. tatricus *and *M. oeconomus *[51-53]) showed no specific V1aR types or AA change corresponding to habitat requirements, nor were they phylogenetically closely related (Figure 3). Additionally, there was no general association of AA variation in *Microtus avpr1a *with social or genetic mating system. The socially monogamous (defined by observational studies, see in [54]) species *M. ochrogaster, M. multiplex *and *M. pinetorum *[51,52,55] shared neither a protein type nor a particular AA change. Similarly, neither showed the socially non-monogamous species such as *M. montanus, M. californicus *and *M. richardsoni *[51,53,56] nor the genetically non- monogamous species (*M. arvalis, M. agrestis and M. ochrogaster*, [18,57-59]) identical AA alterations or protein types among each other that could potentially be associated with behavioural patterns. It is obvious that the high level of variation in the genus *Microtus *makes it very difficult to detect any direct associations of protein types or AA changes with basic life history traits. Since inter- and intra-specific variation in mating behaviour and habitat usage exist (e.g. for *M. ochrogaster *see [17]), studies on protein variation within species are needed to investigate locally adapted receptor types and their correlation to life history traits.

### Evidence for non-neutral evolution

Statistical tests for selection indicated mostly purifying selection on the transmembrane partition of V1aR but also a minor role for positive selection in shaping *avpr1a *diversity in mammals. The sliding window analysis detected positive selection mainly in the ligand binding domain and an increased number of synonymous and non-synonymus changes in the G-protein binding domain. Branch-specific tests across mammals detected positively selected sites mainly in the G-protein binding domain. It is unclear why selection tests failed to detect a deviation from neutrality overall, but the background of purifying selection might be too high in comparison to the positive selection acting on particular domains to allow the signal to be picked up. Additionally, positive selection evidenced by ω > 1 may be difficult to detect as selection could also be acting on synonymous sites [60-62].

The impact of positive selection on *avpr1a *diversity is less evident within the *Microtus *genus than in evolutionarily less related mammalian taxa. Overall selection tests remained mostly inconclusive, which may be caused by a lack of power due to the high rate of speciation and the short divergence times between *Microtus *species [28]. Interestingly, the number of AA variants within *Microtus *was still significantly higher in the ligand and G-protein binding domains than in the transmembrane regions. This pattern might reflect relaxed selective constraints at the N-terminus and at the G-protein binding domain, and stronger evolutionary constraints on the transmembrane region due to structural limitations because of the embedding in the lipid layer. Alternatively, we suggest that this high diversity in functionally important domains of the gene is compatible with balancing selection maintaining high allele diversity in selected regions [63,64]. However, we shall need detailed studies at the population level to asses the potential impact of this type of selection on *avpr1a *diversity in *Microtus *further [65].

### Evolution of *avpr1a *variability in comparison to other genes

The speed of evolutionary change in *avpr1a *is difficult to assess because of the lack of data on nuclear mammalian genes with similar taxonomic and geographic scope. The only directly comparable data set from a nuclear gene covering several *Microtus *species comes from the *p53 *tumor-suppressor gene [66]. Variation in this gene is much lower than in *avpr1a *with only a few silent mutations in coding regions [66]. Additionally, nucleotide diversity in the fast-evolving nuclear genes IRBP and RAG1 within the mouse genus is lower than in Microtine *avpr1a *if the longer divergence time in *Mus *is taken into account (5 to 6 mya for *Mus*, see in [67] vs. 1.2 to 2 mya for *Microtus*, see in [28]). Variation in *avpr1a *appears here even higher after translation of the nucleotide sequences into AAs, because variation in *Mus *is reduced to 9% variable positions in IRBP and 4% in RAG1 [67] whereas 11% variable positions in the AA sequences remain in the vole *avpr1a *gene.

Our comparison of all currently available homologous nuclear genes for the mouse-rat-vole trio showed for the *avpr1a *gene a relatively high synonymous substitution rate but a comparatively low non-synonymous substitution rate (Figure 6). It is unclear to which extent this comparison is somewhat biased by a generally stronger interest and more published sequences of genes with high mutation rates (e. g. MHC, BRCA [68,69]). It is nevertheless noteworthy that this comparison revealed higher nucleotide and protein diversity in *avpr1a *than in other related hormone receptors with similar functions (e.g. oxytocin, see in [16,20,70]; serotonin, see in [71,72]; corticothropin, see in [73,74]).

## Conclusion

Our analyses show that genetic diversity in the *avpr1a *gene is much higher than previously claimed, and that part of this variation might be functionally relevant. We provide evidence for extensive variation in *avpr1a *at all taxonomic levels of mammals, with many changes in functionally important regions. We suggest that positive selection acting on these operative domains helps to maintain variation despite the presence of overall purifying selection. The role of balancing selection, particularly within the genus *Microtus*, should nevertheless deserve further investigation at the intra-specific level. The effects of genetic variation in *avpr1a *on phenotypic traits like mating systems, social behaviour or habitat requirements in *Microtus *and other mammals are far from being characterized. As this study shows, it seems particularly important to characterize abundant genotypic and phenotypic variation thoroughly before establishing general causal links between genotypes and phenotypes.

## Methods

### Samples

The V1aR is encoded by two exons: exon1 (~970 bp) and exon2 (~290 bp). We sequenced part (792 bp) of the first exon of the *avpr1a *gene since this fragment covers the two functionally important regions (ligand and G-protein binding domains) of the receptor. Sequences were analysed for 24 *Microtus *species which cover the entire Palearctic range of the genus (Europe, North America, Asia; Table 1). Tissue samples were obtained by live trapping with Longworth small mammal traps (Penlon Ltd), or from ecologists studying the species. Genomic DNA was extracted using a standard phenol-chloroform protocol [75] or Magnetic beads (MagneSil™ BLUE, Promega). We used two sequences from GenBank from *M. ochrogaster *and *M. montanus *(Accession numbers AF069304 and AF070010) to confirm locus identification.

Moreover, we sequenced three rodent taxa (*Arvicola terrestris, Apodemus sylvaticus and Clethrionomys glareolus*, see Table 1) and retrieved additional mammalian *avpr1a *sequence information from GenBank [76] and Ensembl [77] to compare sequence diversity and substitution rates for the *avpr1a *gene in mammals. Accession numbers in GenBank are: BC024149 for *Mus musculus*, NM_053019 for *Rattus norvegicus*, L41502 for *Ovis aries*, U19906 for *Homo sapiens; *Accession numbers in Ensembl:ENSCAFG00000000339 for *Canis familiaris *ENSBTAG00000007175 for *Bos taurus*, ENSMMUG00000000549 for *Macaca mulatta*, ENSPTRG00000005167 for *Pan troglodytes*, and ENSMODG00000014334 for *Monodelphis domestica*.

### DNA sequencing

We amplified *avpr1a *sequences in a reaction volume of 25 μl in a GeneAmp^® ^PCR System 9700 (Applied Biosystems) using Quiagen *Taq *polymerase. We used two primer pairs for amplification and sequencing reactions: V1aR-5'exon-ProtF 5'-GAGCTTAGGACAGGCTTTCTCG-3' and V1aR-5'exon-ProtR 5'-CGATCACGAAGGTCATCTTCAC-3', Mus-Mic-exon1f 5'-CCGACAGCATGAGTTTCC-3' together with Mus-Mic-exon1r 5'-CCACATCTGGACGATGAAGA-3'. The PCR amplification profile included an initial denaturation step at 92°C for 2 min, followed by 40 cycles of denaturation at 95°C for 1 min, annealing at 55°C for 1 min and extension at 72°C for 90 sec. A final extension step of 72°C for 10 min was performed. Amplified fragments were controlled for size on a 1.5% agarose gel by comparing them with a 100 base pair (bp) ladder (Invitrogen). After cleaning with GenElute™ PCR clean-up kit (Sigma) and dissolving products in 50 μl bi-distilled water, the sequencing reaction was carried out in a 10 μl reaction volume. Terminator Ready Reaction Mix 'Big Dye' Version 3.1 from Applied Biosystems was used. Both strands were sequenced using the following PCR conditions: An initial step of denaturation at 96°C for 10 sec, followed by 30 cycles of denaturation at 96°C for 10 sec, annealing at 55°C for 10 sec, and extension at 72°C for 4 min 30 sec. The products were cleaned using a DyeEx 96 spin kit (Quiagen), and were separated and detected on an ABI Prism 3100 Genetic Analyser from Applied Biosystems.

### Cloning and sequencing of PCR products

PCR products of individuals showing heterozygous sites in direct sequencing were cloned using the Qiagen PCR Cloning Kit. Purified PCR products were quantified in a Spectrophotometer (Gene Quant *pro *RNA/DNA Calculator, Biochrom) and approximately 65 ng of the product were ligated into pDrive Cloning Vector (Qiagen) in 10 μl reactions. Reactions were incubated for 45 min at 4°C before heat shock transformation into QIAGEN EZ Competent Cells. An additional incubation step of 45 min at 37°C with shaking was done before plating to allow recombinant growth. Cells were plated onto Kanamycin-IPTG-X-Gal agar and cultured for 17 h at 37°C. Ten positive clones per individual were randomly selected and further grown in LB broth for 17 h at 37°C with shaking. Plasmid miniprep columns (QIAprep^® ^Spin Miniprep Kit, Qiagen) were used to purify each clone before sequencing with both M13 universal 5'-GTAAAACGACGGCCAGT-3'and M13 reverse 5'-CAGGAAACAGCTATGAC-3' primers. Sequencing conditions were as follows: An initial step of denaturation at 90°C for 50 sec, followed by 25 cycles of denaturation at 90°C for 10 sec, annealing at 50°C for 10 sec, and extension at 60°C for 4 min. After a final cleaning step with a DyeEx 96 spin kit (Quiagen), the sequences were run on an ABI Prism 3100 Genetic Analyser from Applied Biosystems.

### Statistical analyses

Sequences were aligned using the Clustal W algorithm [78] implemented in the program BioEdit 5.0.9 [79], and were revised manually. Shared sequence types were detected using the program Arlequin 3.1 [80]. Phylogenetic relationships among sequenced chromosomes were reconstructed by obtaining neighbour-joining (NJ) [81] and maximum likelihood (ML) trees rooted with *Monodelphis domestica *for the mammalian taxa and rooted with *Arvicola terrestris *for the *Microtus *genus with 10,000 bootstrap replicates in Mega 3 [82] and Paup 4.0 b [83]. For the ML analysis, Modeltest 3.06 [84] implemented in Paup 4.0 b [83] was used to estimate the most suitable model of DNA substitution, by performing hierarchical likelihood ratio tests to compare 52 different models and by applying the Akaike Information Criterion [85]. For the *Microtus *genus, the best substitution model was the transversion model with gamma distribution (TVM+G) with the following parameters: Substitution rate matrix: A↔C 2.7903; A↔G and C↔T 9.3807; A↔T 1.1820; C↔G 0.9720; G↔T 1.0000; and gamma distribution shape parameter 0.1986. The base frequencies were estimated as: A: 0.1801, C: 0.2951, G: 0.2963, T: 0.2285.

For the mammalian phylogeny, the best substitution model was the general time reversible model with invariable sites and gamma distribution (GTR+I+G) [86,87]. The following parameters for the model were estimated: Substitution rate matrix: A↔C 1.7329; A↔G 5.3823; A↔T 0.6124; C↔G 1.4182; C↔T 3.8055; G↔T 1.0000; proportion of invariable sites 0.4474 and gamma distribution shape parameter 3.0860. The base frequencies were estimated as: A: 0.1566, C: 0.3344, G: 0.3228, T: 0.1862.

The nucleotide sequences were translated into AA sequences in Mega 3 using the universal code. The positions of the AA changes were determined using the structural model of the arginine-vasopressin 1a receptor of *Mus musculus *[16]. To determine whether changes are equally distributed across the model, we applied Chi-Square tests for the different structural regions (ligand binding domain, transmembrane regions and G-protein binding domain). AA changes were classified as radical or conservative by comparing physicochemical properties of AAs such as charge, polarity and volume following Zhang [29].

To test for a link between V1aR types and phylogenetic relationships between *Microtus*, we checked for branch specific AA changes of *avpr1a *on a mitochondrial cytochrome *b *gene phylogeny [see in [28]] with sequences obtained from GenBank (accession numbers: AF119280, AF159400, AF163890 –AF163891, AF163893, AF163896, AF163900 –AF163901, AF163903–AF163906, AF187230, AY167210, AY220028, AY220770, AY513788, AY513798, AY513816, AY513819, AY513829, AY513837, AY513840, AY513845). To contrast the synonymous and non-synonymous diversity found in the *avpr1a *gene to other nuclear genes, we performed an exhaustive GenBank search for all annotated gene sequences available for *Microtus *species (up to december 20^th^, 2006). The resulting 31 sequences were aligned with homologous genes from *Mus musculus *and *Rattus norvegicus *and synonymous and non-synonymous substitution rates for each gene were computed with Mega 3.

### Tests for selective neutrality

We tested for regions under positive selection along the mammalian *avpr1a *by estimating the ratio ω of non-synonymous changes (d_N_) over synonymous changes (d_S_) per site. We used a sliding window approach with a window size of 30 and a step size of 10 with the program DnaSP 4.10 to compare mammalian species against the marsupial *Monodelphis domestica*.

To further test for the impact of selection on particular sites in *avpr1a*, we used a maximum likelihood approach with the single likelihood ancestor counting (SLAC) method implemented in HyPhy which makes no assumption about rate variation between lineages [88-90]. Further statistical tests for selection involved the computation of lineage-specific ratios of ω using codon-based maximum likelihood methods implemented in the program "codeml" from the PAML package [91]. As a basis for these analyses, we used a phylogenetic tree tested for consistent topology between ML and NJ as well as with data from 3^rd ^codon positions only [see [92]].

We used likelihood ratio tests in PAML to compare different neutral (MO, M1, M7) and selection (M2, M8) models of DNA sequence evolution of *avpr1a*. In all these tests, two times the log-likelihood difference (2Δ*l*) between models is compared to a χ^2 ^distribution with the number of degrees of freedom (dF) equal to the difference in the number of parameters between the models [93]. We tested for rate heterogeneity among lineages by comparing the one ratio model M0 against the discrete model M3 where different rates are allowed [93]. This test is mainly used to check for rate variation of ω, but it can also be used to detect positive selection [94]. Additionally, the neutral model M1 with two ratio classes of ω (< 1 and 1) was compared to the selection model M2 which allows for an additional class where ω > 1 [93]. A similar comparison was carried out between a neutral model assuming a beta distribution of ω (M7), and a model with similar characteristics but allowing for positively selected sites (M8) [93]. We performed branch specific tests to examine whether *avpr1a *evolves differently in the higher mammalian taxa by comparing the neutral model M1 with model MA which allows for positively selected sites on a pre-selected branch [91,94].

## Competing interests

The author(s) declares that there are no competing interests.

## Authors' contributions

GH and LE conceived this study. SF performed the molecular work and the statistical analyses. All authors discussed the results. SF and GH wrote the paper, and all authors commented on it and approved the final version of the manuscript.
